# Whether the Golgi protein 73 could be a diagnostic serological marker in hepatocellular carcinoma: a meta analysis

**DOI:** 10.1186/s12876-023-02685-8

**Published:** 2023-03-24

**Authors:** Xu Zhang, Li-Na Wu, Xiao-Qing Li, Xia Luo, Shui-Wei Liu, Le Zhang, Shah Nawaz, Li-Na Ma, Xiang-Chun Ding

**Affiliations:** 1 Department of Infectious Disease, General Hospital of Ningxia Medical University, Yinchuan, 750004 Ningxia China; 2 Ningxia Medical University, Yinchuan, Ningxia China; 3 Department of Infectious Disease, The 2nd Affiliated Hospital of Chengdu Medical College Nuclear Industry 416 Hospital, Chengdu, Sichuan China

**Keywords:** Golgi protein 73 (GP73), Hepatocellular carcinoma (HCC), Cirrhosis, Diagnostic, Meta-analysis

## Abstract

**Background:**

The Value of Golgi protein 73 (GP73) in the diagnosis of Hepatocellular carcinoma (HCC) remains controversial, especially in its differentiation between HCC and cirrhosis. Besides, some papers showed that GP73 levels are correlated with liver fibrosis. This study conducts a meta-analysis to evaluate the value of GP73 in diagnosing HCC and differential diagnosing HCC from liver cirrhosis.

**Methods:**

36 studies with a sample size of 8314 cases concerning the accuracy of GP73 in the diagnosis of HCC were selected through a systematic review. Seven of these studies included a total of 438 HCC samples and 426 cirrhosis samples and calculated the sensitivity and specificity of GP73 for differential diagnosing HCC from cirrhosis. QUADAS (quality assessment of diagnostic accuracy studies) was used to evaluate the quality of literature. Statistical analyses were performed using StataSE16 software.

**Results:**

The pooled sensitivity, specificity, positive likelihood ratio, negative likelihood ratio, diagnostic odds ratio and the area under the curve were 0.79(95%CI 0.74–0.83),0.85(95%CI 0.80–0.89),5.4(95%CI 3.8–7.5), 0.25(95%CI 0.20–0.31), 22(95%CI 13–35), and 0.88 for GP73 diagnosing HCC;0.74(95%CI 0.64–0.81),0.70(95%CI 0.49–0.85),2.40(95%CI 1.3–4.7),0.38(95%CI 0.23–0.61),6(95%CI 2–19), and 0.78 for GP73 differential diagnosing HCC from liver cirrhosis.

**Conclusion:**

The results suggest that GP73 has a high diagnostic value for HCC and a moderate value for differential diagnosis of HCC from liver cirrhosis.

## Background

Hepatocellular carcinoma (HCC) is the second leading cause of male cancer death globally, the fourth common malignant tumor, and the third leading cause of cancer death in China. HCC accounts for 85-90% of primary liver cancer, seriously threatening people’s lives and health security [1–3]. HCC high-risk groups, including cirrhosis caused by various reasons [3]. Early diagnosis and treatment of HCC are essential to obtain better therapeutic effects and reduce medical costs [4]. However, due to the insidious onset of HCC and the lack of typical symptoms in the early stage, early monitoring and screening of high-risk groups such as severe hepatitis and liver cirrhosis are particularly important.

In the past 40 years, alpha-fetoprotein (AFP), as a unique HCC-specific serum marker, has been widely used in detecting, diagnosing, evaluating the treatment effect, and predicting recurrence of HCC and has played an important role. Still, 30% of patients with HCC do not show increased AFP, and even sometime it can be negative, which increases the diagnosis difficulty of HCC [5]. To date, many protein markers, such as AFP-L3, IL6 and PIVKA-II, have also been conducted to varying degrees [6]. However, their accuracy could not meet people’s expectations for the early diagnosis of HCC, according to the 2018 global cancer statistics, there are 841,080 new cases and 781,631 deaths of liver carcinoma were reported [7].Besides, the incidence of liver cancer is snowballing compared to other types of cancer on the basis of American cancer statistics in 2020 [8]. Consequently, the situation of HCC patients is still rigorous. Thus, it is imperative to continue to look for new HCC-specific tumor markers.

Golgi protein 73 (GP73), a GolgiII type membrane protein (GOLPH2 / GOLM1), was found in recent years. It is more likely to express in normal colon, lung, kidney, prostate epithelial cells and the bile duct epithelial cells of the normal liver, but not express in normal liver cells [9]. Studies have shown that serum levels of GP73 in HCC patients were significantly higher than those in patients with other severe liver diseases such as cirrhosis and healthy controls [10, 11], indicate that GP73 may be a potential serum marker in the diagnosis of HCC. However, there have been several studies examining serum GP73 as a tumor marker for HCC with conflicting results, many studies suggest that GP73 is not useful in the diagnosis of HCC, and the serum GP73 level of patients with liver cirrhosis is even higher than that of HCC [12–15].Because of the above controversies, we must make a systematic meta-analysis of the relevant literature on GP73 diagnosing HCC.

## Methods

Two researchers searched relevant Chinese and English literature published in Cochrane Library, Pubmed, CNKI, EMBASE, and Wan fang database from January 2014 to January 2022. The key words included “Golgi protein 73/GP73/GOLPH2/GOLM1”, “Hepatocellular carcinoma/ HCC” both in Chinese and English. To improve the recall rate, we conduct a retrospective search from the references of relevant literature. For the retrieved literature, the ones that did not meet the standards were firstly excluded according to the title. Then the literature that met the requirements were screened by reading the abstract. After careful reading, the full text, and the inclusion and exclusion criteria were combined to determine the included literature.

Inclusion criteria: (1) the diagnostic criteria for HCC clearly described in the literature. Patients diagnosed with HCC according to the criteria taken as the experimental group and patients with other liver diseases and health examiners as the control group. (2) Serum GP73 determined in all samples. (3) The true positive, false positive, false negative and true negative values of GP73 for the diagnosis of HCC could be obtained directly or indirectly from literature to list 2 × 2 table. (4) The published literature in Chinese or English. Exclusion criteria: (1) non-diagnostic studies. (2) Incomplete data could not list 2 × 2 table. (3) Repeated publication.

Relevant data collected from the selected literature, including author, study year, country, sample content (number of cases in the experimental group and the control group), sample characteristics (age and gender distribution), GP73 detection method, specificity, and sensitivity, etc. And true positive, false positive, false negative and true negative values were calculated. QUADAS (quality assessment of diagnostic accuracy studies) was used to evaluate the quality of literature.

The meta-analysis of diagnostic tests in StataSE16.0 software was used. We drew the forest chart of the pooled sensitivity, specificity, positive likelihood ratio (PLR), negative likelihood ratio (NLR), diagnostic odds ratio (DOR) of the random effect model were calculated. Summary receiver operating characteristic curve (SROC) was drawn using the appropriate data statistical model, and we calculate the area under the curve (AUC).Discuss the threshold effect and the heterogeneity was tested. The Funnel Plot was plotted using theStataSE16.0 software for detecting publication bias.

## Results

### Study selection and study-quality analysis

**Fig. 1:**
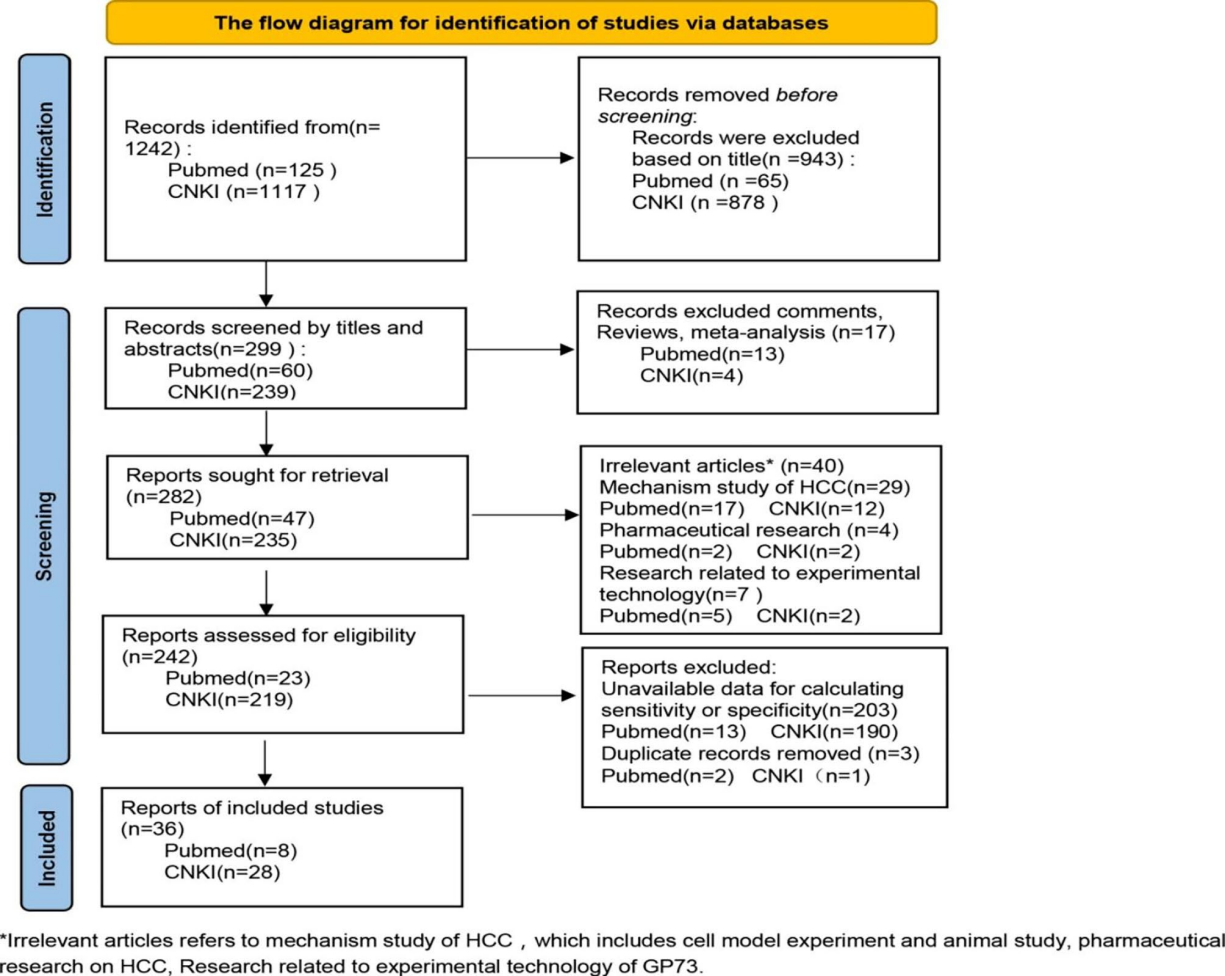
Flow diagram of the process of the inclusion and exclusion of studies for this meta-analysis

The study recruitment flowchart is shown in Fig. 1. We included 36 studies [15–50]with a total sample size of 8314 cases. Among them, HCC patients with or without cirrhosis accounted for 3192 cases, and the remaining 5122 cases with non-HCC included cirrhosis, benign liver tumors, and non-liver tumors and healthy people. Seven of these studies had 438 HCC samples and 426 cirrhosis samples in which the sensitivity and specificity of GP73 for differential diagnosing HCC from cirrhosis were calculated [15, 29, 37, 44–47]. Table 1 shows the characteristics of the 36 included studies of studies, including some extract relevant data( the country of publication, sample size, gender, age, specificity, sensitivity, etc.). Meanwhile, the quality evaluation of the included literature was conducted according to the QUADAS scale. The scores were all above 10, indicating the relatively high quality of the selected literature.

**Table 1:** Characteristics of the included studies

Author	Year	Country	HCC/ Control	Age	Gender (M/F)	method	Sensitivity (%)	Specificity(%)	TP	FP	FN	TN	AUC	QUADAS
Sun BB [46]	2022	China	20/20	38–70	25/15	ELISA	70	90	14	2	6	18	——	11
Liu YM [45]	2018	China	50/140	25–81	109/81	ELISA	64	68.57	32	44	18	96	0.865	10
			50/50	25–81	58/42	ELISA	64	56	32	22	18	28		12
NZekri AR [43]	2020	Egypt	78/160	27–74	154/84	ELISA	91	85	71	24	7	136	0.956	11
Eissa M [39]	2020	Egypt	25/62	34–64	59/28	ELISA	88	87	22	8	3	54	0.924	10
Farag RMA [41]	2019	Saudi	145/105	23–78	185/65	ELISA	95	95	138	13	7	98	0.896	13
			145/105	23–78	185/65	ELISA	100	90	145	95	0	10	——	11
Li XY [43]	2020	China	12/55	——	47/20	ELISA	83.33	69.1	8	15	4	40	0.758	10
Jiao C [38]	2017	China	180/263	——	333/110	ELISA	78.3	85.5	141	38	39	225	0.84	11
Shaker MK [40]	2020	Egypt	32/96	20–73	76/52	ELISA	96.87	96.87	31	3	1	93	0.969	12
Wang ZY [49]	2022	China	66/66	45–75	67/65	ELISA	77.4	67.4	51	3	15	44	——	10
Wang XM [48]	2019	China	82/241	39–76	248/75	ELISA	71.95	93.78	59	15	23	226	——	11
Gao Y [42]	2020	China	80/68	——	88/60	ELISA	70	77.94	56	53	24	15	0.971	12
Zhou ZJ [47]	2020	China	65/73	——	75/63	ELISA	86.61	91.78	55	6	10	67	——	11
Zhang DQ [50]	2020	China	148/271	——	176/243	ELISA	76.3	83.8	113	44	35	227	0.635	11
Bo L [15]	2017	China	34/75	35–83	66/43	ELISA	47.10	69.30	16	23	18	52	0.539	14
ManarM I [16]	2017	Saudi Arabia	66/83	16–82	89/60	ELISA	90.90	97.60	60	2	6	81	0.963	11
Huang WZ [17]	2017	China	117/80	29–78	137/60	ELISA	82.05	81.25	96	15	21	65	——	10
Qian HG [18]	2017	China	50/150	29–68	146/54	ELISA	96.00	93.33	48	10	2	140	——	10
Wang XY [19]	2017	China	120/217	47–79	215/122	ELISA	78.60	88.90	94	24	26	193	——	10
Liu MH [20]	2016	China	40/40	41–78	45/35	ELISA	77.50	85.00	31	6	9	34	——	10
Guo M [21]	2016	China	40/105	21–68	85/60	ELISA	90.00	91.43	36	9	4	96	——	10
Gao G [22]	2015	China	194/166	51.47	287/73	ELISA	65.50	66.30	127	56	67	110	0.713	13
Liu X [23]	2015	China	69/279	48.74	183/165	ELISA	73.80	86.80	51	37	18	242	0.816	10
Zhao Y [24]	2015	China	50/100	32–81	98/52	ELISA	72.00	94.00	36	6	14	94	0.826	12
Zhao SY [25]	2015	China	68/117	23–74	99/86	ELISA	80.88	97.44	55	3	13	114	——	10
Zhang Q [26]	2015	China	86/88	25–80	122/52	ELISA	63.95	72.72	55	24	31	64	——	10
Xu WJ [27]	2014	China	50/145	46.49	128/67	ELISA	80.00	97.20	40	4	10	141	0.841	10
Mirelle E [28]	2014	Netherlands	88/176	19–82	66/198	ELISA	60.00	77.00	53	40	35	136	0.701	13
Wang Y [29]	2014	China	84/173	50.91	186/71	Western blot	73.60	81.50	62	32	22	141	0.88	11
			84/80	54.49	129/35		82.10	80.00	69	16	15	64	0.92	
Zhang HJ [30]	2014	China	145/314	24–83	275/184	ELISA	71.00	85.40	103	46	42	268	——	10
Zhao Y [31]	2014	China	50/80	21–81	88/42	ELISA	72.00	95.00	36	4	14	76	0.824	10
Xu H [32]	2014	China	81/127	51.86	161/47	ELISA	48.10	74.00	39	33	42	94	0.704	10
Jia HL [33]	2014	China	74/67	32–78	99/42	ELISA	75.68	91.04	56	6	18	61	0.811	12
Zhang FH [34]	2014	China	50/150	35–76	149/51	ELISA	80.00	97.20	40	4	10	146	0.8411	10
Guo W [35]	2014	China	105/130	56.58	179/56	ELISA	77.14	72.38	81	36	24	94	——	10
Zhao Y [36]	2014	China	59/56	——	105/10	ELISA	64.40	96.40	38	2	21	54	0.852	10
Xu QM [37]	2014	China	105/302	20–77	292/115	ELISA	80.00	75.17	84	75	21	227	——	11
			105/60	22–77	111/54		80.00	58.33	84	25	21	35	——	

HCC:Hepatocellular Carcinoma;M:Male;F:Female;TP:True positive;FP:False positive; FN:False negative;TN:True negative;AUC:the area under the receiver operating characteristic curves;QUADAS:quality assessment of diagnostic accuracy studies

### Summary diagnostic value of GP73

**Fig. 2:**
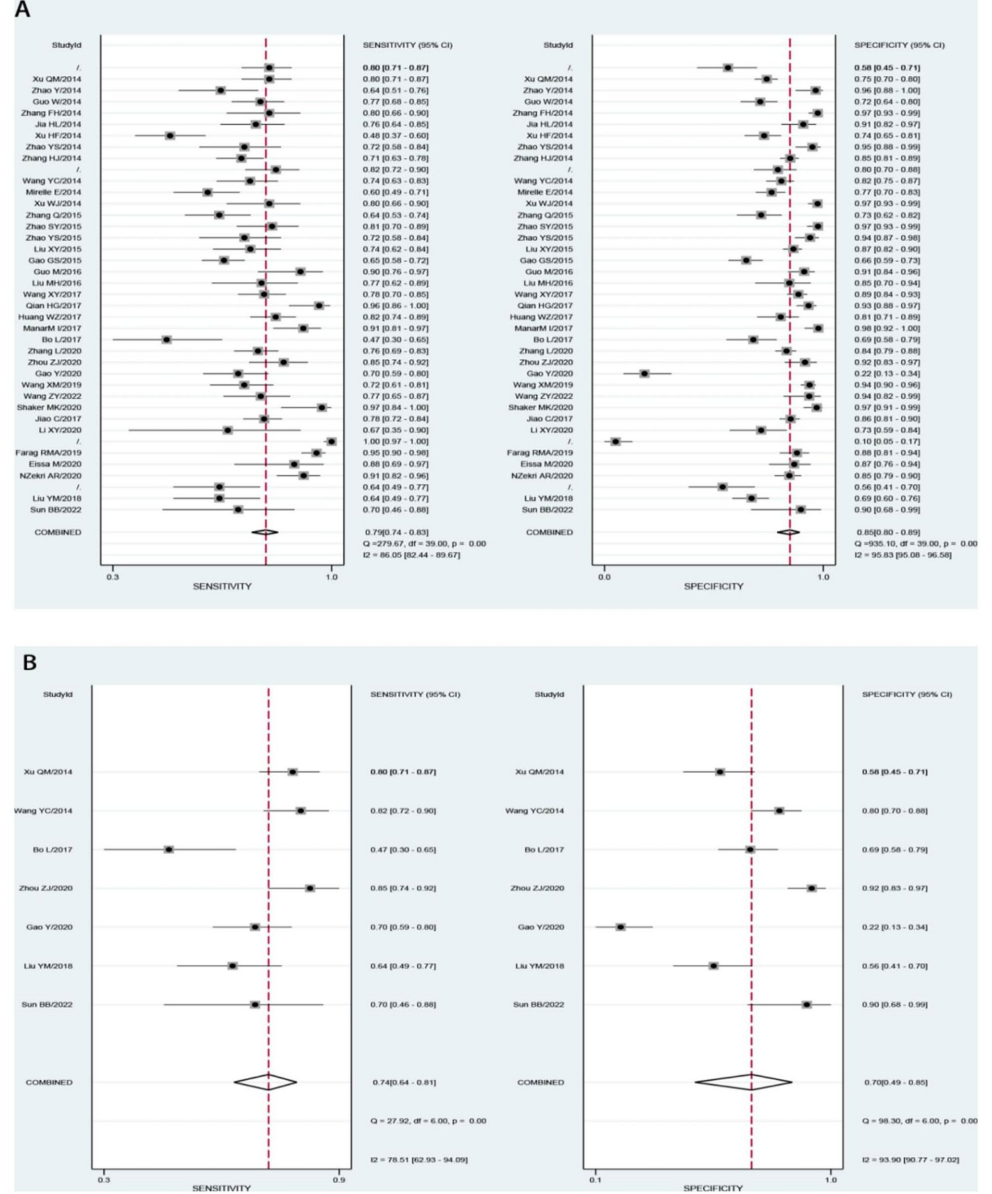
Pooled sensitivity and specificity (A) for GP73 diagnosing HCC and (B) for GP73 differential diagnosing HCC from cirrhosis

**Fig. 3:**
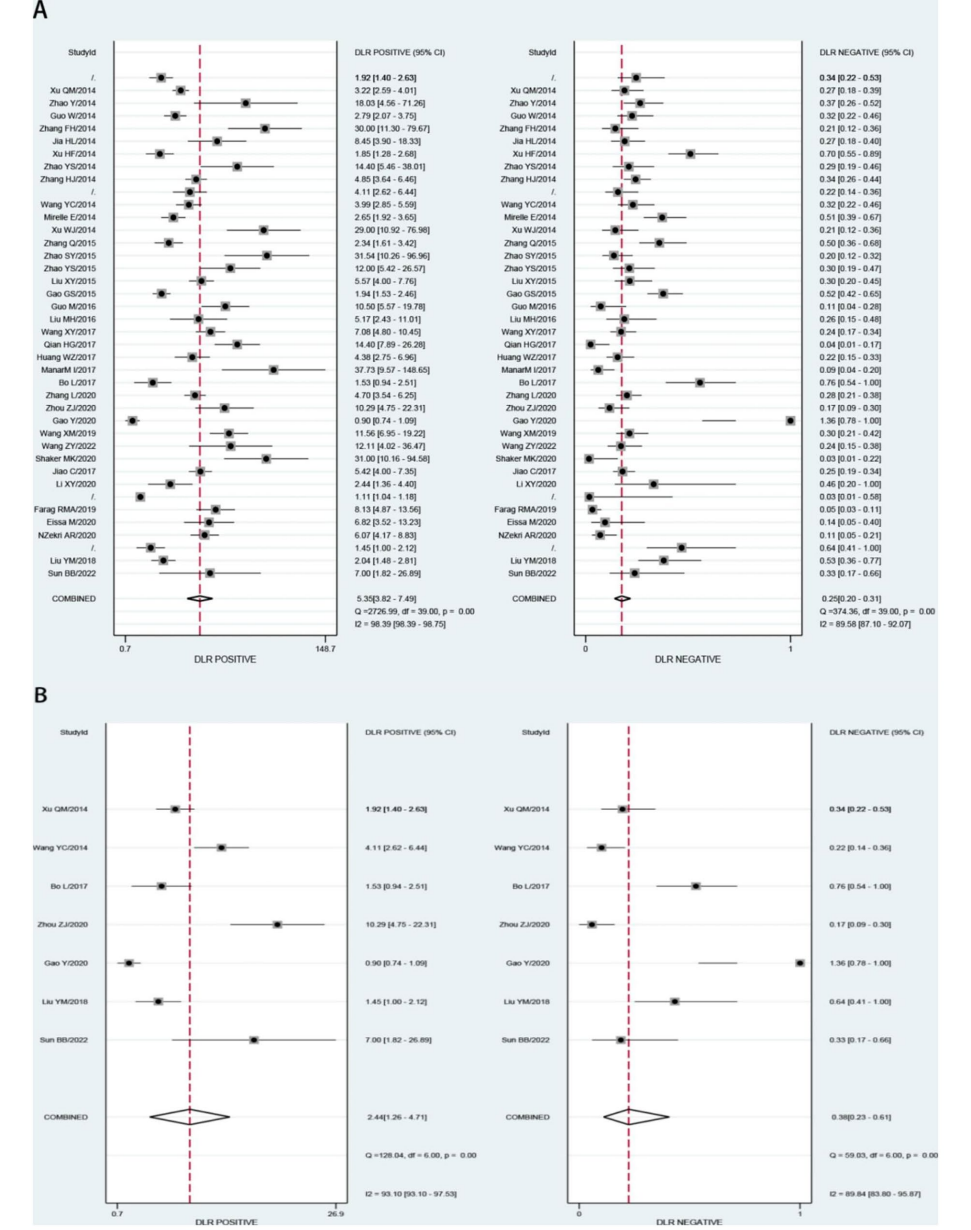
PLR and NLR for GP73 diagnosing HCC and (B) for GP73 differential diagnosing HCC from cirrhosis

**Fig. 4:**
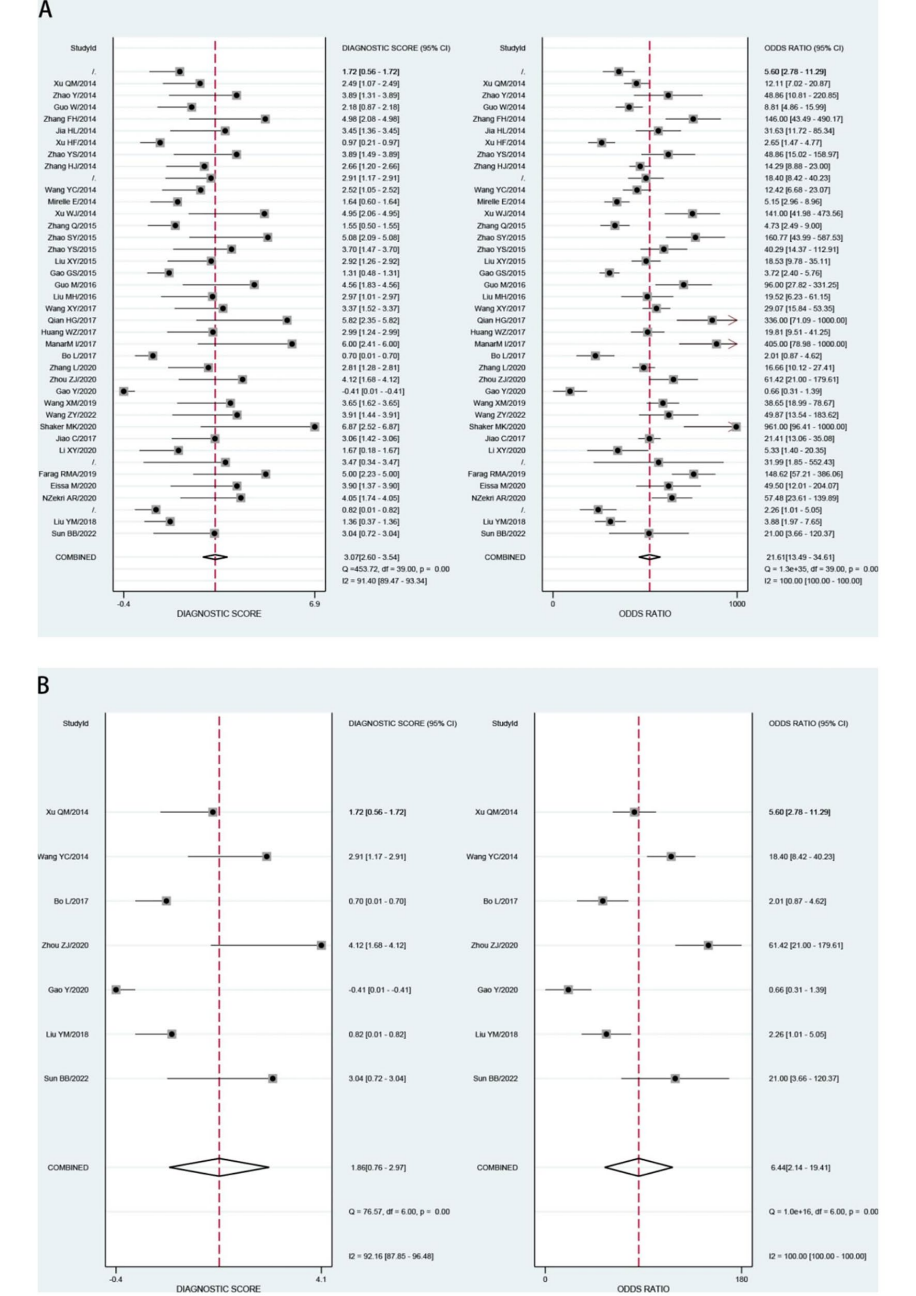
DOR (A) for GP73 diagnosing HCC and (B) for GP73 differential diagnosing HCC from cirrhosis

**Fig. 5:**
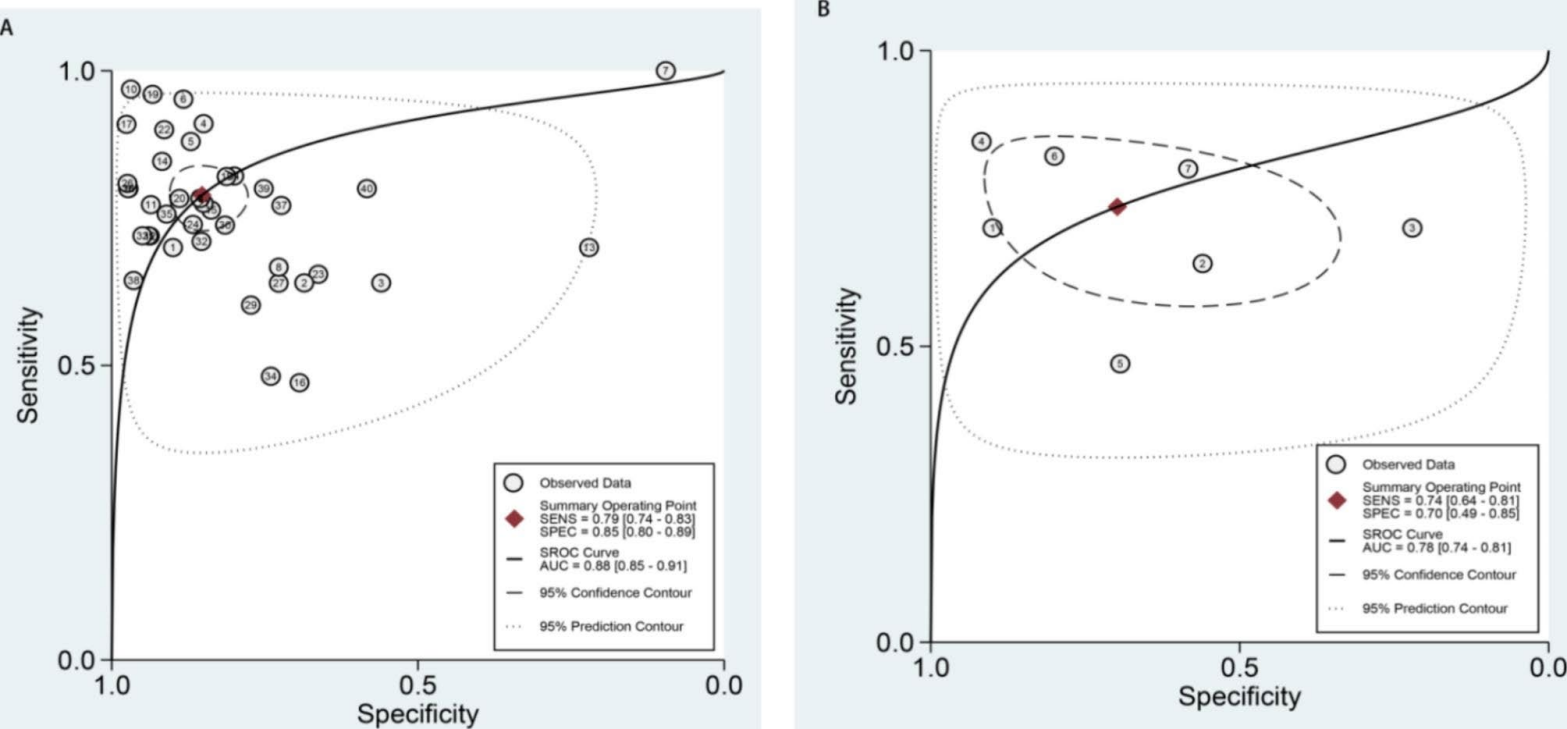
SROC curve(A) of GP73 diagnosing HCC and (B) of GP73 differential diagnosing HCC from cirrhosis

**Fig. 6:**
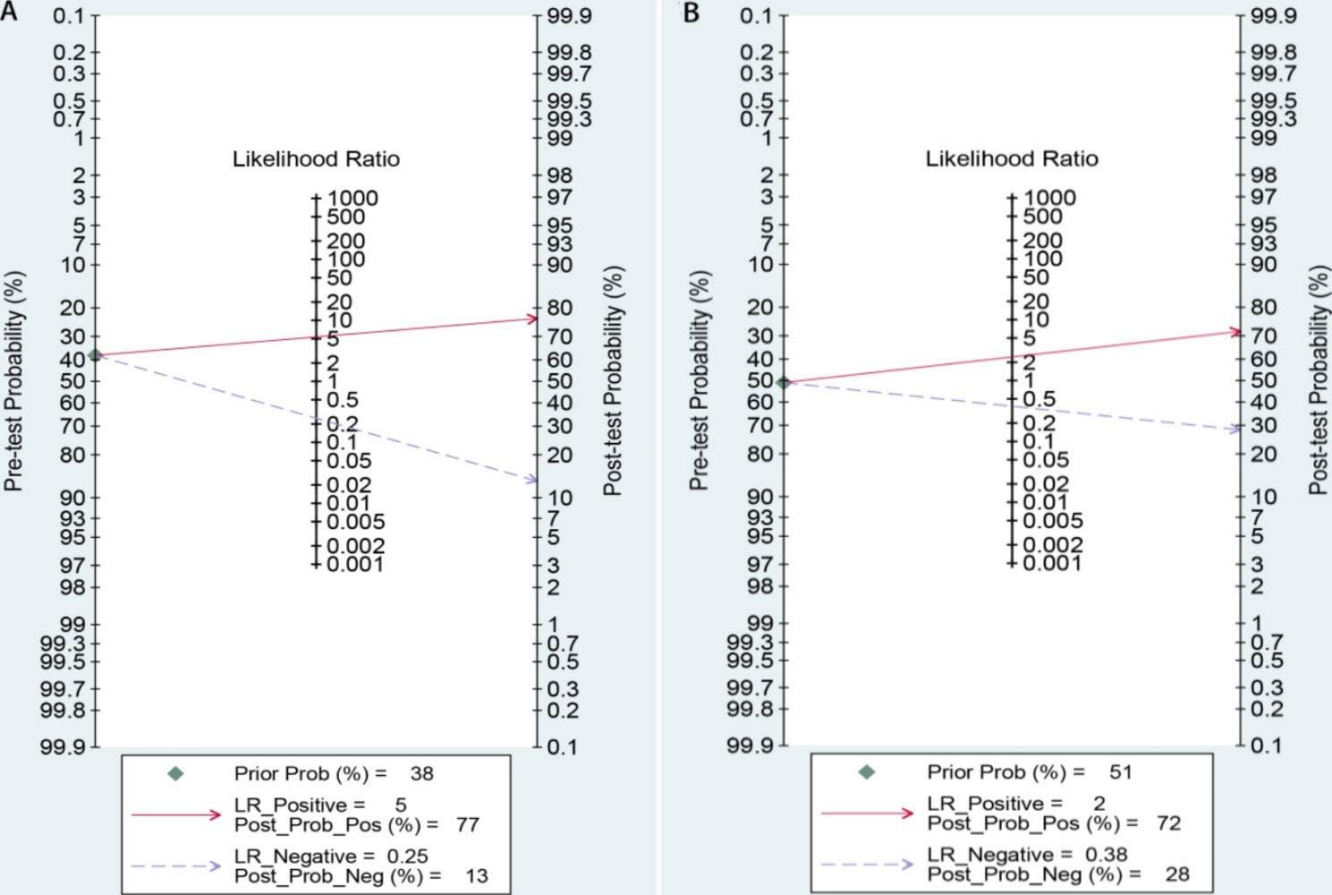
Fagan nomogram (A) for GP73 diagnosing HCC and (B) for GP73 differential diagnosing HCC from cirrhosis

We analyzed the value of GP73 diagnosing HCC and GP73 differential diagnosing HCC from cirrhosis in our study with the different control groups. Both analyses sensitivity and specificity showed high heterogeneity, so the random effect model was chosen to combine the effect size.The Table 2 displays the accuracy of GP73 in diagnosis of HCC (the large-sample Group) and GP73 in differential diagnosis of HCC and cirrhosis(the cirrhosis Group). The pooled sensitivity of GP73 diagnosing HCC from the control group of large samples, including healthy controls was 0.79, and the 95% confidence interval (CI) was 0.74–0.83. The heterogeneity test showed that I^2^ = 86.05%. The pooled specificity was 0.85, with a 95% CI of 0.80–0.89, I^2^ = 95.83%(Fig. 2A). The pooled PLR was 5.35, with a 95% CI of 3.82–7.49, I^2^ = 90.3%. The pooled NLR was 0.25, with a 95% CI of 0.20–0.31, I^2^ = 89.58% (Fig. 3A). The pooled DOR was 21.61, with a 95% CI of 13.49–34.61, I^2^ = 100% (Fig. 4A). The fitted SROC curve is shown in Fgure 5 A. The AUC is 0.88, with a 95% CI of 0.85–0.91. Also, the pooled sensitivity of GP73 diagnosing HCC from cirrhosis samples as the control group was 0.74, and the 95% CI was 0.64–0.81, and the heterogeneity test showed I^2^ = 78.51%. The pooled specificity was 0.70, and the 95% CI was 0.49–0.85, I^2^ = 93.90% (Fig. 2B). The pooled PLR was 2.44, with a 95% CI of 1.26–4.71, I^2^ = 93.1%. The pooled NLR was 0.38, with a 95% CI of 0.23–0.61, I^2^ = 89.84%(Fig. 3B). The pooled DOR was 6.44, with a 95% CI of 2.14–19.41, I^2^ = 100% (Fig. 4B).The fitted SROC curve was shown in Fig. 5B. The AUC is 0.78, with a 95% CI of 0.74–0.81. In addition, the Fagan nomogram revealed that the post-test propability of the GP73 diagnosing HCC was 77% and GP73 differential diagnosing HCC from cirrhosis was72%, indicating GP73 identified highly valuable in diagnosing HCC or diagnosing HCC differential from cirrhosis (Fig. 6).

**Table 2:** Summary of the diagnostic accuracy of GP73

Group	Pooled sensitivity(95%CI)	Pooled specificity(95%CI)	Pooled PLR(95%CI)	Pooled NLR(95%CI)	Pooled DOR(95%CI)	AUROC (95%CI)
Large-sample Group	0.79 (0.74–0.83)	0.85 (0.80–0.89)	5.35 (3.82–7.49)	0.25 (0.20–0.31)	21.61 (13.49–34.61)	0.88 (0.85–0.91)
Cirrhosis Group	0.74 (0.64–0.81)	0.70 (0.49–0.85)	2.44 (1.26–4.71)	0.38 (0.23–0.61)	6.44 (2.14–19.41)	0.78 (0.74–0.81)

PLR:positive likelihood ratio;NLR:negative likelihood ratio;DOR:diagnostic odds ratio;AUROC:the area under the receiver operating characteristic curve; CI: Confidence interval.

### Test for heterogeneity

In this meta-analysis, large heterogeneity was observed and the reasons for heterogeneity were investigated. In this meta-analysis, Firstly,meta-regression method was performed to explore the heterogeneity with published period, country, sample size,GP73 detection method as the co-variate to analyze possible reasons for the heterogeneity,the results are shown in Table 3.We discovered the published period, country and sample size may be the causes of heterogeneity of the pooled sensitivity, while **s**ample size and GP73 detection method may be the reasons for heterogeneity of the pooled specificity. Additionally, the causes of heterogeneity should explore the proportion of heterogeneity likely due to threshold effect is zero, suggesting that the heterogeneity isn’t caused by the threshold effect.

**Table 3:** Meta-regression analysis of the pooled sensitivity and specificity

Parameter	Category	NO.studies	95%CI of Sensitivity	P	95%CI of Specificity	P
period	Before 2017	26	0.769(0.71–0.81)	0.00	0.88(0.83–0.93)	0.16
	After 2017	14	0.84(0.78–0.90)		0.80(0.69–0.90)	
country	China	33	0.75(0.71–0.79)	0.00	0.86(0.80–0.91)	0.17
	Not China	7	0.92(0.88–0.96)		0.84(0.71–0.96)	
Sample size	>60	25	0.79(0.74–0.85)	0.00	0.82(0.75–0.89)	0.00
	≤ 60	15	0.78(0.70–0.85)		0.90(0.85–0.96)	
method	ELISA	37	0.90(0.82–0.98)	0.70	0.87(0.82–0.91)	0.00
	other	3	0.78(0.73–0.82)		0.56(0.24–0.88)	

NO.studies:Number of studies;CI:Confidence interval.

### Publication bias

**Fig. 7:**
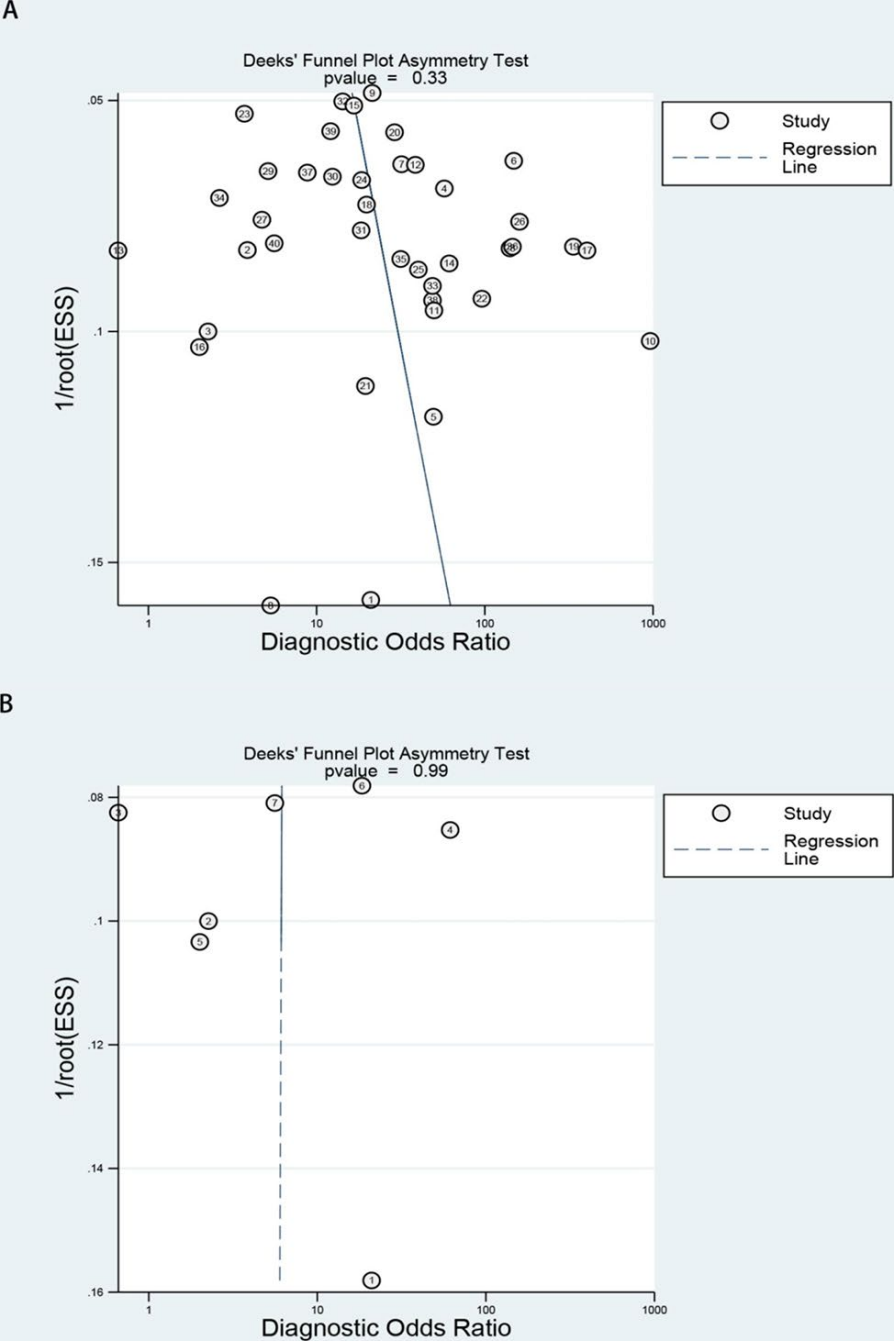
The Deeks^,^ funnel plots to assess potential publication bias (A) for GP73 diagnosing HCC and (B) for GP73 differential diagnosing HCC from cirrhosis

In the publication bias test, we used the StataSE16 software to draw the funnel diagram as shown in Fig. 7, indicating no bias.

## Discussion

Hepatocellular carcinoma is a global disease. Its early diagnosis plays a vital role in improving the prognosis of patients and saving social resources. Because the low sensitivity of AFP is becoming increasingly difficult to meet the needs of early diagnosis of HCC, people begin to continually look for new tumor markers.

Since Phillips et al. found the cDNA clone fragment of GP73 in patients with CMV hepatitis; GP73 has been closely associated with liver diseases [9]. In 2005, Block et al. reported for the first time that in animal liver cancer cells GP73 is highly expressed, and in human patients with HCC the serum level of GP73 is significantly increased [16]. Meanwhile, Marrero et al. showed that serum GP73 level in patients with HCC was significantly higher than that in patients with liver cirrhosis (p < 0.001). The sensitivity and AUC for GP73 early diagnosing HCC were both higher than AFP, suggesting that GP73 may become a serum marker for early diagnosis of HCC [15].

Subsequently, studies on GP73 related to HCC have been published one after another. Up to now, a large number of studies on the diagnostic Value of GP73 for HCC have been reported, as well as number of meta-analyses [51–53]. In 2015, Dai’s meta-analysis [51] including 11 studies showed that GP73 had a sensitivity of 0.77 and specificity of 0.91 in the diagnosis of HCC and a DOR of 12.49, which were better than AFP. Still, its AUC of 0.86 was less than 0.91, the combination of GP73 and AFP. In 2020, the meta-analysis of Zhao et al. [19] showed that GP73 had a sensitivity of 0.77 and specificity of 0.93 in the diagnosis of HCC and a DOR of 43, which were better than AFP. However, its AUC of 0.90 was less than 0.95, when combined the Golgi protein 73, glypican-3 and AFP. Moreover, Zhang et al. [53] included 9 studies indicated that GP73 over expression was significantly associated with later tumor stage, higher tumor grade and poor overall survival (OS).

In brief, most studies have shown that GP73 could be used as a potential serum marker for the diagnosis of HCC. However, still at the same time, many studies have also shown that there was no statistically significant difference in the expression level of serum GP73 between patients with HCC and cirrhosis. Even the serum GP73 of patients with liver cirrhosis is higher than that of patients with HCC, suggesting that GP73 cannot distinguish HCC from liver cirrhosis^54^–^56^ Liu et al.‘s research in 2017 showed that the AUROC of 0.613 in the differential diagnosis of HCC from cirrhosis had a noticeable decrease than 0.834 in the differential diagnosis of HCC from other chronic liver diseases. Moreover, GP73 levels had no noticeable change after the resection of HCC lesions, which were different from AFP declining significantly. The authors believed that GP73 could not accurately distinguish HCC patients from non-HCC patients with cirrhosis [14].Because of this controversy, we collected relevant literature to conduct a meta-analysis to evaluate the diagnostic value of GP73 for HCC with varying groups of control. One contained patients with various non-HCC diseases and healthy individuals; the other included patients with liver cirrhosis alone.

Our meta-analysis included a total of 36 studies. Among them, Bo et al. [13] followed up 109 patients with liver cirrhosis for 36 months and compared serum GP73 levels of patients who turn to liver cancer or not. Sun  [46], Gao  [42], Zhou  [47] evaluated the diagnostic Value of GP73 for HCC with a control group of patients with liver cirrhosis, Wang et al. [29], Xu et al. [37] and Liu et al. [45]evaluated the diagnostic value of GP73 for HCC with a control group of patients with hepatitis, liver cirrhosis, other benign liver diseases, and healthy people. They then evaluated it in patients with liver cirrhosis as the control group.

The remaining 29 studies were all GP73 diagnostic studies of HCC with the control group of other liver diseases, tumors at different sites, and healthy people. Due to the high heterogeneity of the pooled sensitivity and specificity (I^2^ was 86.05% and 95.83%, respectively), we selected the random effect model for statistical analysis. The pooled sensitivity and specificity were 0.79 and 0.85, slightly lower than Dai et al.‘s study [51] in 2015 with the sensitivity of 0.77 and specificity of 0.91. The change of the etiological spectrum of HCC might be one factor.

We drew the fitted SROC curve, the AUC was 0.88, showed GP73 has better diagnostic value for HCC. The positive likelihood ratio (PLR) indicates that patients with HCC are 5.35 times more likely to be correctly diagnosed as positive than non-HCC patients. The negative likelihood ratio (NLR) indicates that patients with HCC are 0.25 times less likely to be wrongly diagnosed as negative. Diagnostic odds ratio (DOR) is an index to evaluate the performance of a diagnostic test. It integrates the accuracy of sensitivity and specificity and is the ratio of PLR to NLR. Its value can range from 0 to infinity. The larger the value is, the better the diagnostic efficiency will be. The DOR of 21.61 indicates that GP73 has high diagnostic efficacy for HCC.

In addition, we also calculated the diagnostic value of GP73 for HCC in the seven studies [15, 29, 37, 44–47] from which only taking liver cirrhosis as the control group, and we obtained that the pooled sensitivity and specificity were 0.74 and 0.70, with the AUROC of 0.78. It indicates that GP73 has a moderate value for the differential diagnosis of HCC from cirrhosis, but it is lower than that of the former analysis. The DOR of 6.44 also shows less effectiveness than the former. Hence, one can see that GP73 has a relatively moderate ability of differential diagnosis between HCC and cirrhosis.

This may be explained by Liu et al. [57]. Their study showed that hepatoma cells and activated hepatic stellate cells could express GP73 in patients with liver disease. In contrast, the hepatic stellate cells in patients with liver cirrhosis are largely activated to express high levels of GP73, which might indicate the close association between the two groups.

In this meta-analysis, the heterogeneity test of the pooled sensitivity and specificity both showed I^2^ > 50%, indicating high heterogeneity. Therefore, the reasons for heterogeneity were investigated. Firstly, meta-regression method was performed to explore the heterogeneity according to the studies’ characteristics in the former meta-analysis (Table 3), we discovered the published period, country and sample size may be the causes of heterogeneity of the pooled sensitivity, while sample size and GP73 detection method may the reasons for heterogeneity of the pooled specificity. However, the regression analysis cannot be conducted due to the small sample size in the latter analysis. Additionally, no threshold effect was detected from the SROC curve. As we can see, it have statistically significant effect caused by sample size, publication period and country in terms of diagnostic accuracy, we speculate that some causes might include the different types resulting from hepatitis and the heterogeneous control group comprising cirrhosis, benign liver tumors and non-liver tumors, and healthy people proportion differences. We drew the Deeks’ funnel plot for quality evaluation, both of them indicating no bias which may caused by data errors, improper use of statistical methods, failure to include a large number of studies, true heterogeneity or other factors.

## Conclusion

In conclusion, our study shows that GP73 has a relatively high efficiency for diagnosing HCC, and it also has a moderate value for differential diagnosing HCC from liver cirrhosis. But precisely how GP73 is expressed in liver tissues and cells of cirrhotic patients remains to be studied.

## Data Availability

The datasets used during the current study are available from the corresponding author on reasonable request.

## List of Abbreviations

GP73: Golgi protein 73
HCC: Hepatocellular Carcinoma
PLR: Positive likelihood ratio
NLR: Negative likelihood ratio
DOR: Diagnostic odds ratio
SROC: Summary receiver operating characteristic
AUC: Area under the curve
CI: Confidence interval

## References

[1] Ruman U, Fakurazi S, Masarudin MJ, Hussein MZ (2020). Nanocarrier-Based therapeutics and Theranostics Drug Delivery Systems for Next Generation of Liver Cancer Nanodrug Modalities. Int J Nanomedicine.

[2] Ding Y, Xie Q, Liu W, Pan Z, Fan X, Chen X (2020). Neochamaejasmin A induces mitochondrial-mediated apoptosis in human hepatoma cells via ROS-Dependent activation of the ERK1/2/JNK signaling pathway. Oxid Med Cell Longev.

[3] Shrestha R, Prithviraj P, Anaka M, Bridle KR, Crawford DHG, Dhungel B (2018). Monitoring Immune Checkpoint regulators as predictive biomarkers in Hepatocellular Carcinoma. Front Oncol.

[4] Bou-Nader M, Caruso S, Donne R, Celton-Morizur S, Calderaro J, Gentric G (2020). Polyploidy spectrum: a new marker in HCC classification. Gut.

[5] Wang Q, Chen Q, Zhang X, Lu XL, Du Q, Zhu T (2019). Diagnostic value of gamma-glutamyltransferase/aspartate aminotransferase ratio, protein induced by vitamin K absence or antagonist II, and alpha-fetoprotein in hepatitis B virus-related hepatocellular carcinoma. World J Gastroenterol.

[6] Pang YY, Li JD, Gao L, Yang X, Dang YW, Lai ZF (2020). The clinical value and potential molecular mechanism of the downregulation of MAOA in hepatocellular carcinoma tissues. Cancer Med.

[7] Bray F, Ferlay J, Soerjomataram I, Siegel RL, Torre LA, Jemal A (2018). Global cancer statistics 2018: GLOBOCAN estimates of incidence and mortality worldwide for 36 cancers in 185 countries. CA Cancer J Clin.

[8] Zhu W, Peng Y, Wang L, Hong Y, Jiang X, Li Q (2018). Identification of alpha-fetoprotein-specific T-cell receptors for hepatocellular carcinoma immunotherapy. Hepatology.

[9] Gatselis NK, Tornai T, Shums Z, Zachou K, Saitis A, Gabeta S (2020). Golgi protein-73: a biomarker for assessing cirrhosis and prognosis of liver disease patients. World J Gastroenterol.

[10] Wu M, Liu Z, Li X, Zhang A, Li N (2020). Dynamic changes in serum markers and their utility in the early diagnosis of all stages of Hepatitis B-Associated Hepatocellular Carcinoma. Onco Targets Ther.

[11] Zhang Z, Zhang Y, Wang Y, Xu L, Xu W (2016). Alpha-fetoprotein-L3 and golgi protein 73 may serve as candidate biomarkers for diagnosing alpha-fetoprotein-negative hepatocellular carcinoma. Onco Targets Ther.

[12] Hu B, Tian X, Sun J, Meng X (2013). Evaluation of individual and combined applications of serum biomarkers for diagnosis of hepatocellular carcinoma: a meta-analysis. Int J Mol Sci.

[13] Li B, Li B, Guo T, Sun Z, Li X, Li X (2017). The clinical values of serum markers in the early prediction of Hepatocellular Carcinoma. Biomed Res Int.

[14] Liu T, Yao M, Liu S, Wang L, Wang L, Hou J (2017). Serum golgi protein 73 is not a suitable diagnostic marker for hepatocellular carcinoma. Oncotarget.

[15] Ye JZ, Yan SM, Yuan CL, Wu HN, Zhang JY, Liu ZH (2018). GP73 level determines chemotherapeutic resistance in human hepatocellular carcinoma cells. J Cancer.

[16] Ismail MM, Morsi HK, Abdulateef NAB (2017). Evaluation of prothrombin induced by vitamin K absence, macrophage migration inhibitory factor and golgi protein-73 versus alpha fetoprotein for hepatocellular carcinoma diagnosis and surveillance. Scand J Clin Lab Investig.

[17] Huang W, Zhou F, Pan D (2017). Diagnostic valueof serum golgi protein combined with Dickkopf-1 and alpha-fetoprotein for hepatocellular carcinoma. Guangdong Med J.

[18] Qian H, Liu C, Hu Y (2017). The role of GP73 and AFP Detection in the diagnosis of Hepatocellular Carcinoma. Int J Lab Med.

[19] Wang X (2017). Diagnostic value of combined detection of serum GP73, AFP and AFP-L3 in primary hepatocellular carcinoma. Labeled immunoassays and Clinical Medicine.

[20] Liu M, Yao L (2016). Application of combined detection of serum AFP, AFP-L3 and GP73 in the diagnosis of primary hepatocellular carcinoma. China Continuing Medical Education.

[21] Mei G, Lin X, Jin Z (2016). Value of alpha fetoprotein heterogeneity and golgi protein 73 in the diagnosis of primary hepatocellular carcinoma. J Clin Med Pract.

[22] Gao G, Dong F, Xu X (2015). Diagnostic value of serum golgi protein 73 for HBV-related primary hepatic carcinoma. Int J Clin Experimental Pathol.

[23] Liu X, Wan X, Lu S et al. Lu.Evaluation of a magnetic particles-based chemiluminescence enzyme immunoassay for Golgi protein 73 in human serum. Clinica chimica acta; international journal of clinical chemistry,2015,445:54 – 9. 10.1016/j.cca.2015.03.008 25801213

[24] Zhao Y, Wang M, Cui C, et al. Significance of combined tests of serum golgi glycoprotein 73 and other biomarkers in diagnosis of small primary hepatocellular carcinoma. Volume 15. Labeled Immunoassays & Clinical Medicine; 2016. pp. 677–83. 5. 10.3233/CBM-150508 PMC12965448 26406957

[25] Shu-Yan Z, Huang-Dao Q (2015). The diagnostic value of combined detection of AFP, AFP-L3 and GP73 for primary hepatocellular carcinoma [J]. Chin J Lab Diagnosis.

[26] Qin Z (2015). Value of serum GP73, AFP, and AFP-L3 in diagnosis of liver cancer and recurrence monitoring after radiofrequency ablation[J]. J Clin Hepatol.

[27] Xu WJ, Guo BL, Han YG (2014). RETRACTED ARTICLE: diagnostic value of alpha-fetoprotein-L3 and golgi protein 73 in hepatocellular carcinomas with low AFP levels[J]. Tumor Biology.

[28] Mirelle EE, Bröker, Ijzermans JNM, Witjes CDM (2014). The predictive value of golgi protein 73 in differentiating Benign from Malignant Liver Tumors[J]. PLoS ONE.

[29] Wang Y, Yang H, Xu H (2014). Golgi protein 73, not Glypican-3, may be a tumor marker complementary to α-Fetoprotein for hepatocellular carcinoma diagnosis.[J]. J Gastroenterol Hepatol.

[30] Zhang H, Ning S, Wang J et al. Value of Golgi Protein 73 Quantitative Detection in the Diagnosis of Hepatocellular Carcinoma. Shandong Medical Journal, 2014 (33):57–58.

[31] Zhao Y, Wang M, Cui C et al. Evaluate the clinical Value of eight serum markers in the diagnosis of primary liver cancer [J].Chongqing Medicine, 2014 (2):214–216.

[32] Xu H, Wang L, Zhu C (2014). The role of combined detection of serum GP73, AFP, AFP-L3 and AFU in the diagnosis of primary hepatocellular carcinoma [J]. Anhui Med Pharm J.

[33] Jia H, Huang C, Song J (2014). Diagnostic value of combined detection of serum alpha-fetoprotein variant, golgi protein 73 and glutamyl transpeptidase in hepatocellular carcinoma [J]. Cancer Res Clin.

[34] Zhang F, Li J (2014). The value of combined detection of alpha-fetoprotein variant and golgi protein 73 in the diagnosis of Hepatocellular Carcinoma [J]. Chin J Practical Med.

[35] Guo W (2014). Value of three serum tumor markers in the diagnosis of primary hepatocellular carcinoma [J]. Experimental and Laboratory Medicine.

[36] Zhao Y, Li Y, Hu B (2014). The role of golgi protein 73 in the diagnosis and prediction of hepatocellular carcinoma [J]. Natl Med J China.

[37] Xu Q, Chen H, Duan Z (2014). Diagnostic value of GP73 combined with AFP and GGT-Domain detection in primary hepatocellular carcinoma [J]. Mod Med J China.

[38] Jiao C, Cui L, Piao J, et al. Clinical significance and expression of serum golgi protein 73 in primary hepatocellular carcinoma. J Cancer Res Ther. 2018 Oct-Dec;14(6):1239–44. 10.4103/0973-1482.199784 30488837

[39] Eissa M, Awad S, Barakat S, et al. Serum golgi protein 73 as a sensitive biomarker for early detection of hepatocellular carcinoma among egyptian patients with hepatitis C virus-related cirrhosis. Med J Armed Forces India. 2021 Jul;77(3):331–6. 10.1016/j.mjafi.2020.11.013 PMC8282533 34305287

[40] Shaker MK, Attia FM, Hassan AA et al. Evaluation of Golgi Protein 73 (GP73) as a Potential Biomarkers for Hepatocellular Carcinoma.Clin Lab. 2020 Aug 1;66(8). 10.7754/Clin.Lab.2020.190911 32776730

[41] Farag RMA, Al Ayobi D, Alsaleh KA et al. Studying the Impact of Golgi Protein 73 Serving as a Candidate Biomarker in Early Diagnosis for Hepatocellular Carcinoma among Saudi Patients.Asian Pac J Cancer Prev. 2019 Jan25;20(1):215–220. 10.31557/APJCP.2019.20.1.215 PMC6485586 30678434

[42] Zekri N, El Kassas AR, Salam M, Hassan ESE, Mohanad RM, Gabr M, Lotfy RMM, Abdel-Zaher M, Bahnassy RAT, Ahmed AA. OS. The possible role of Dickkopf-1, Golgi protein- 73 and Midkine as predictors of hepatocarcinogenesis: a review and an Egyptian study. Sci Rep. 2020 Mar 20;10(1):5156. 10.1038/s41598-020-62051-6 PMC7083902 32198440

[43] Li XY, Han TT,etal. Xia TianDiagnostic value of AFP-L3 and GP73 in primary liver cancer[J].Guide of China Medicine, 2020,18(07): 3–4. DOI:10.15912/j.cnki.gocm.2020.07.003

[44] Y Gao. The difference of HBV DNA, AFP-L3, GP73 and the correlation between HBV DNA and AFP-L3, GP73 in patients with hepatitis B cirrhosis and liver cancer[J].Laboratory Medicine and Clinic,2020,17(07):938–941.

[45] YM Liu S, He. Y Cao,etal.Diagnostic value of GP73,AFP-L3,TIP30 combined detection in liver cirrhosis caused by hepatitis B[J].International Journal of Laboratory Medicine,2018,39(22):2794–2800.

[46] BB Sun (2022). Clinical value of AFP,GPC3,GP73,CA125 combined examination in the diagnosis of primary liver cancer[J]. Mod Diagnosis Treat.

[47] Zhou ZJ, Yuan F. Clinical value of combined examination of AFP, GPC3, GP73 and CA125 in patients with primary liver cancer[J].Journal of Jilin Medical College,2020,41(02):95–97.DOI:10.13845/j.cnki.issn1673-2995.2020.02.006.

[48] XM Wang. Application value of combined detection of serum DCP, GP73 and AFP-L3 levels in patients with primary liver cancer[J].nner mongolia medical journal,2019,51(06):720–722.DOI:10.16096/J.cnki.nmgyxzz.2019.51.06.040.

[49] Wang Zhiyuan ZY, Lu LQ, Niu JH (2022). Diagnostic value of combined detection of serum AFP, AFP-L3, AFU and GP73 in primary liver cancer[J]. Hainan Med J.

[50] Zhang DQ, Zhang HG. XR Zhang,etal.Clinical value of combined detection of serum AFP, PIVKA-II, GP73 and IL-6 in primary liver cancer[J].Chinese Journal of coal industry medicine,2022,25(02):212–216.

[51] Dai M, Chen X, Liu X, Peng Z, Meng J, Dai S (2015). Diagnostic value of the combination of golgi protein 73 and alpha-fetoprotein in Hepatocellular Carcinoma: a Meta-analysis. PLoS ONE.

[52] Zhang J, Zhang M, Ma H, Song X, He L, Ye X (2020). A meta-analysis of the prognostic significance of golgi protein 73 in hepatocellular carcinoma in chinese patients. Arch Med Sci.

[53] Zhao S, Long M, Zhang X, Lei S, Dou W, Hu J (2020). The diagnostic value of the combination of golgi protein 73, glypican-3 and alpha-fetoprotein in hepatocellular carcinoma: a diagnostic meta-analysis. Ann Transl Med.

[54] Norton PA, Comunale MA, Krakover J, Rodemich L, Pirog N, D’Amelio A (2008). N-linked glycosylation of the liver cancer biomarker GP73. J Cell Biochem.

[55] Yao M, Wang L, Leung PSC, Li Y, Liu S, Wang L (2018). The clinical significance of GP73 in immunologically mediated Chronic Liver Diseases: Experimental Data and Literature Review. Clin Rev Allergy Immunol.

[56] Gatselis NK, Zachou K, Giannoulis G, Gabeta S, Norman GL, Dalekos GN. Serum Cartilage Oligomeric Matrix Protein and Golgi Protein-73: New Diagnostic and Predictive Tools for Liver Fibrosis and Hepatocellular Cancer?Cancers (Basel). 2021;13(14). 10.3390/cancers13143510 PMC8304371 34298722

[57] Liu MY, Huang L, Wu JF, Zhang HB, Ai WB, Zhang RT (2022). Possible roles of golgi protein-73 in liver diseases. Ann Hepatol.

